# Kinematic analysis of forelimb and hind limb joints in clinically healthy sheep

**DOI:** 10.1186/s12917-014-0294-4

**Published:** 2014-12-12

**Authors:** Luis G Faria, Sheila C Rahal, Felipe S Agostinho, Bruno W Minto, Lídia M Matsubara, Washington T Kano, Maira S Castilho, Luciane R Mesquita

**Affiliations:** Department of Veterinary Surgery and Anesthesiology, School of Veterinary Medicine and Animal Science – Univ Estadual Paulista (UNESP), Botucatu, SP Brazil; Department of Clinic and Veterinary Surgery, Faculdade de Ciências Agrárias e Veterinárias – UNESP, Jaboticabal, SP Brazil

## Abstract

**Background:**

Variations associated with sex, age, velocity, breed and body geometry should be considered in the determination of kinematic parameters for a gait considered normal.

Therefore, this study aimed to evaluate kinematic patterns of forelimbs and hind limbs in clinically normal sheep from two different age groups walking at a constant velocity. The hypothesis was that the age may influence sagittal plane kinematic patterns. Fourteen clinically healthy female sheep were divided into Group 1 – seven animals aged from 8 to 12 months, and Group 2 - seven animals aged above 5 years. Before starting data collection, the sheep were trained to be conducted for walking in a pre-determined space at constant velocity. A minimum of 5 valid trials were obtained from the right and left sides of each sheep. Data were analyzed by use of a motion-analysis program. Flexion and extension joint angles (maximum, minimum, displacement), and angular velocity (maximum, minimum) were determined for the shoulder, elbow, carpal, hip, stifle, and tarsal joints.

**Results:**

Within each group, no significant differences were observed between the right and left limbs in all kinematic variables. Significant differences were observed in the following kinematic parameters between G1 and G2: minimum angle (G1 < G2), angular displacement (G1 > G2), maximum angular velocity (G1 > G2), minimum angular velocity (G1 > G2) of the carpus; angular displacement (G1 > G2), minimum angular velocity (G1 > G2) of the shoulder; minimum angle (G1 > G2), angular displacement (G1 < G2) of the tarsus; maximum angular velocity (G1 < G2) of the stifle; maximum angular velocity G1 > G2 of the hip. The lengths of both forelimbs and hind limbs differed between groups (G1 < G2). The Froude number differed between groups for forelimbs and hind limbs.

**Conclusions:**

In conclusion, sheep of two different ages walking at a constant velocity present, within the same group, similar kinematic data between sides, and exhibit some differences in kinematic variables that may be age-related or body size. Further studies using sheep walking at similar Froude numbers are necessary to exclude the body size.

## Background

Several instrumentation types are available for kinematic evaluation including films, video recordings, television/computer, and optoelectronic systems that present considerable differences in terms of convenience and accuracy [1,2]. Most gait-analysis laboratories use a computer system to collect the data, in which markers placed at strategic locations on the body, or pre-determined anatomical landmarks, have the trajectories captured by specialized cameras [2,3].

Kinematic gait analysis can be used to evaluate healthy individuals or individuals with diseases [1,4-7]. However, to enhance understanding of the abnormal gait requires determination of parameters for a gait considered normal; "normal" should be interpreted by taking into account variations associated with sex, age and body geometry [1,8]. In addition, morphological variations associated with the breeds should be considered in animals [3,6,9].

The stages of musculoskeletal growth and maturation of the central nervous system play an important role in gait analysis [1,10]; the walking pattern in an adult human is obtained at the age of 7 years, and gait parameter differences become stable at approximately 16 to 18 years old [11]. In addition, changes of locomotion may occur with advancing age. For example, elderly people may have altered excursion of joint movement, such as reduction in the total range of hip flexion and extension, in swing phase knee flexion, and in ankle plantar flexion [1].

Some kinematic studies have used the sheep as the experimental model [12-14]. Merino-mix sheep were used to evaluate the soft tissue coverage in the ascertainment of bone kinematics by means of skin-mounted markers [12]. Tridimensional stifle kinematics was applied to quantify in Suffolk-cross sheep the influence of the complete lateral meniscectomy [13]. Kinematic abnormalities measured by 3D stifle kinematic were correlated with degrees of early osteoarthritis in surgical models of anterior cruciate ligament/medial collateral ligament transection performed in Suffolk-cross sheep [14].

Therefore, the purpose of the present study was to evaluate kinematic patterns of forelimbs and hind limbs in clinically normal sheep from two different age groups walking at a constant velocity. The hypothesis was that age may influence sagittal plane kinematic patterns.

## Methods

This study was approved by the Ethics Committee of School of Veterinary Medicine and Animal Science – Univ Estadual Paulista (UNESP) (no. 42/2011-CEUA). Fourteen clinically healthy intact female sheep, client owned, all of the Santa Ines breed, were used: seven animals aged from 8 to 12 months and weighing 19–33 kg (G1), and seven animals aged more than 5 years and weighing 37–45 kg (G2). The owner of the sheep gave his consent to perform the experiment.

The animals were judged to be healthy on account of results of complete physical and orthopedic examinations. Before starting the kinematic analysis, the sheep were trained to be conducted for walking in a pre-determined space at constant velocity by the same handler. Approximately seven days before the recordings, hoof trimming was accomplished.

### Data collection

Kinematic analysis was performed using a 5-camera system (T10S camera - NIR 12.5; Vicon, Peak Performance Technologies Inc, Colorado, USA). For each analysis, the system was calibrated and a three-dimensional testing space (3 m in length × 2.5 m in width × 2 m in height) was established.

Each sheep was tagged with 11 retroreflective spherical markers (1.8 cm in diameter) by a single investigator, as previously described [6]. Markers were placed on the skin using quick-drying glue over the dorsal point of the iliac crest, lateral prominence of the ischial tuberosity, greater trochanter of the femur, femorotibial joint between the lateral epicondyle of the femur and the fibular head, lateral malleolus, distal lateral aspect of metatarsi III and IV, the point of the cranial angle of the scapula, acromion of the scapulohumeral joint, lateral epicondyle of the humerus, styloid ulnar process, and distal lateral aspect of metacarpi III and IV.

A minimum of 5 valid trials were obtained and analyzed first from the left side, then the right side of each sheep; each trial included a complete stride cycle. Trials were considered valid if the animal walked within the predetermined velocity and acceleration, and without head movement or pulling on the halter. Data were analyzed by use of a motion-analysis program (Vicon Nexus). The velocity was maintained 1.1-1.3 m/s and acceleration from −0.15 to 0.15 m/s^2^ determined by a pressure-sensitive walkway (Walkway High Resolution HRV4; Tekscan, South Boston, Massachusetts, USA).

The 11 individual markers were identified and labeled to construct a 3D stick-diagram representation of the sheep. The maximum, minimum and displacement values were obtained from normalized trials of each animal. A stride was defined from the beginning of the stance phase of one limb to the end of its swing phase. For the hind limb the beginning of the stance phase/swing phase was determined by the inversion moment of angular velocity of the hip joint at the end of each respective phase. For the forelimb, the beginning of stance phase/swing phase was determined by the inversion moment of angular velocity of the elbow joint at the end of each respective phase.

Flexion and extension joint angles (maximum, minimum, displacement), and angular velocity (maximum, minimum) were determined for the shoulder, elbow, carpal, hip, stifle, and tarsal joints. The length of each limb was established by the sum of the distances between each pair of markers on that limb.

The Froude number was calculated for both forelimbs and hind limbs as follow: Fr = v^2^/gl (v = velocity, g = acceleration due gravity, l = limb length) [15].

### Statistical method

To compare kinematic parameters between the right and left limbs within the same group, and data between the groups, one-way ANOVA was used followed by Tukey’s *post-hoc* test. The values were expressed as mean ± standard deviation, and the coefficients of variation (CV) were calculated. An independent sample *t* test was used to compare lengths of the forelimbs and hind limbs and Froude number between groups. Differences were considered significant at *P* < 0.05.

## Results

Within each group, no significant differences were observed between the right and left limbs in all kinematic variables. Significant differences were observed in the following kinematic parameters between G1 and G2: minimum angle (G1 < G2), angular displacement (G1 > G2), maximum angular velocity (G1 > G2), minimum angular velocity (G1 > G2) of the carpus; angular displacement (G1 > G2), minimum angular velocity (G1 > G2) of the shoulder; minimum angle (G1 > G2), angular displacement (G1 < G2) of the tarsus; maximum angular velocity (G1 < G2) of the stifle; maximum angular velocity G1 > G2 of the hip (Tables 1 and 2).

**Table 1:** **Comparison of maximum angle (°), minimum angle (°), displacement angular velocity (°), maximum angular velocity (°/s), and minimum angular velocity (°/s) of the forelimb joints between Group 1 and Group 2**

**Variable**		**Group 1**		**Group 2**		
		**Mean ± SD**	**CV**	**Mean ± SD**	**CV**	***P*** **value**
Carpal	Maximum angle	176.03 ± 3.20	1.82	174.89 ± 2.44	1.40	0.296
	Minimum angle	105.12 ± 4.71	4.48	116.52 ± 10.82	9.28	**0.002**
	Angular displacement	70.91 ± 3.58	5.05	58.37 ± 9.97	17.08	**<0.001**
	Maximum angular velocity	794.57 ± 110.34	13.89	512.37 ± 84.47	16.49	**<0.001**
	Minimum angular velocity	−845.58 ± 139.72	−16.52	−630.36 ± 108.99	−17.29	**<0.001**
Elbow	Maximum angle	150.84 ± 3.38	2.24	149.97 ± 4.58	3.05	0.571
	Minimum angle	106.93 ± 5.13	4.80	107.94 ± 10.68	9.90	0.755
	Angular displacement	43.91 ± 4.64	10.56	42.04 ± 8.73	20.76	0.484
	Maximum angular velocity	416.46 ± 67.64	16.24	401.42 ± 73.60	18.33	0.578
	Minimum angular velocity	−363.90 ± 60.62	−16.66	−331.36 ± 54.89	−16.56	0.149
Shoulder	Maximum angle	134.04 ± 5.92	4.42	131.60 ± 6.68	5.07	0.316
	Minimum angle	121.62 ± 5.20	4.28	122.15 ± 7.20	5.89	0.826
	Angular displacement	12.41 ± 2.32	18.72	9.45 ± 2.19	23.17	**0.002**
	Maximum angular velocity	196.21 ± 74.01	37.72	147.64 ± 48.38	32.77	0.050
	Minimum angular velocity	−197.97 ± 73.54	−37.15	−110.87 ± 23.07	−20.81	**0.001**

*P*-values in bold represent significant differences (P < 0.05) between the mean of G1 and G2 variables.

**Table 2:** **Comparison of maximum angle (°), minimum angle (°), displacement angular velocity (°), maximum angular velocity (°/s), and minimum angular velocity (°/s) of the hind limb joints between Group 1 and Group 2**

**Variable**		**Group 1**		**Group 2**		
		**Mean ± SD**	**CV**	**Mean ± SD**	**CV**	***P*** **value**
Tarsal	Maximum angle	156.48 ± 3.91	2.50	154.59 ± 5.13	3.32	0.470
	Minimum angle	126.44 ± 5.54	4.38	119.30 ± 5.93	4.97	**0.003**
	Angular displacement	30.05 ± 3.99	13.27	35.30 ± 4.45	12.59	**0.003**
	Maximum angular velocity	329.52 ± 72.66	22.05	360.03 ± 58.18	16.16	0.633
	Minimum angular velocity	−336.94 ± 61.62	−18.29	−340.06 ± 54.45	−16.01	0.991
Stifle	Maximum angle	156.48 ± 6.95	5.11	130.59 ± 6.82	5.22	0.084
	Minimum angle	103.13 ± 5.55	5.38	95.60 ± 8.48	8.87	**0.010**
	Angular displacement	32.85 ± 4.73	14.41	34.99 ± 4.88	13.95	0.426
	Maximum angular velocity	313.96 ± 53.66	17.09	363.16 ± 43.15	11.88	**0.026**
	Minimum angular velocity	−217.28 ± 39.58	−18.22	−204.37 ± 34.44	−16.85	0.590
Hip	Maximum angle	156.48 ± 9.01	8.38	107.63 ± 5.52	5.12	0.999
	Minimum angle	89.22 ± 6.26	7.02	91.81 ± 5.32	5.79	0.426
	Angular displacement	18.30 ± 7.32	40.00	15.82 ± 2.79	17.65	0.399
	Maximum angular velocity	213.79 ± 139.77	65.37	126.15 ± 42.16	33.42	**0.031**
	Minimum angular velocity	−169.91 ± −65.53	−38.57	−131.28 ± 17.03	−12.98	0.050

*P*-values in bold represent significant differences (P < 0.05) between the mean of G1 and G2 variables.

The lengths of both forelimbs (*P* = 0.008) and hind limbs (*P* < 0.001) differed between groups (G1 < G2). The differences were approximately 10 cm in forelimbs and 9 cm in hind limbs. The Froude number differed between groups (*P* < 0.001) for forelimbs (G1 = 0.28 ± 0.013; G2 = 0.24 ± 0.016) and hind limbs (G1 = 0.27 ± 0.012; G2 = 0.24 ± 0.025).

## Discussion

Normative studies have been carried out to characterize joint movement patterns in healthy dogs during trotting [6,16-22] or walking [23-25], which are considered symmetrical gaits due to the reciprocity of forelimb as well as hind limb movements [2,9]. In the present study each sheep’s velocity was maintained from 1.1 to 1.3 m/s, which has been previously reported as walking locomotion [6,26].

Kinematic studies showed that to avoid interference, prior to data collection, some dog breeds require multiple training sessions [21] and others not [20]. In sheep studies it must also be considered that these animals become distressed due to changes in the social environment, especially when separated from the rest of the flock [27-29]. Thus, it is important that the sheep be halter-trained before obtaining kinematic data, as performed in the present study.

Although studies employing inverse dynamics have shown that the Labrador retriever dog breed may present a dominant side [30,31], sagittal kinematic studies in dogs did not detect significant differences between right and left sides in forelimbs or hind limbs suggesting symmetry [6,19]. Likewise, in the present study no statistical differences were observed between right and left limbs in all the kinematic parameters. In addition, studies using a pressure-sensitive walkway found that kinetic and temporospatial parameters did not differ between sides [6,26].

In the forelimbs of both groups, the carpal joint had the highest angular displacement value, followed by the elbow joint, with the shoulder presenting the lowest value. In the hind limbs, the tarsal and stifle joints showed similar angular displacements, while the hip joint had the lowest value. Despite differing values, kinematic data from healthy dogs trotting on a treadmill showed similar patterns of sequence of angular displacement, for both forelimb and hind limbs [7,19]. A kinematic study that evaluated gait abilities of two lamb crossbreeds in three different environments also detected that the amplitude of angular variation rose gradually toward the distal extremity of the forelimbs and hind limbs [32]. On the other hand, a two-dimensional kinematic study in healthy dogs during walking found little variation in angular displacement values of the hind limb joints [24]. However, sheep perform stiff walk with hind limbs and compliant walk with forelimbs while dogs present a stiff walk for both forelimbs and hind limbs [33].

With regard to angular velocity of the hind limbs, in both groups the tarsal joint produced the highest value, followed by the stifle joint, while the hip joint had the lowest value. In a study using measures of nonlinear dynamics in trotting dogs, it was observed that the hip joint also presented the lowest angular velocity but that the stifle joint showed the greatest value [17]. In the forelimbs of both groups, the carpal joint ranked highest, followed by the elbow joint, whereas the shoulder had the lowest value. Since the angular velocity corresponds to the rate of change in angular displacement with respect to elapsed time [1,34,35], the data suggest that forces generated during locomotion in sheep require higher angular velocities for more distal joints related to increased angular displacement.

Humans may present a decrease in joint motion with advancing age [1] that has been associated with cross-linkage development between adjacent collagen fibrils and a decrease in the muscle mass, among others [36]. In the present study significant differences were observed in forelimbs in relation to angular displacement of the carpus (G1 > G2) associated with minimum angle (G1 < G2), and angular displacement (G1 > G2) of the shoulder. The data suggest that these joints display greater flexion-extension motion in younger sheep than in older ones probably due to the former’s higher flexibility. In addition, higher angular velocity of the carpus was observed in G1 indicating that flexion and extension rates were faster in this group. Because this group had shorter forelimbs and hind limbs, and all animals walked at the same velocity, the data could suggest a compensation due to their different sizes [37]. The Froude number confirmed that limb length differences occurred between groups. However, to obtain the same Froude number the G1 animals would have to walk 0.1 m/s more slowly than G2. To have this control over the velocity, the animal should be walking on a treadmill.

On the other hand, significant differences were observed in angular displacement of the tarsal joint (G1 < G2) associated with minimum angle (G1 > G2). In a study to establish predictive performance values of trotting horses, despite the kinematic differences based on height differences, it was also reported that during growth, the joint angles become more extended [38], which may have influenced the necessity of lesser angular displacement with higher tarsal flexion in G1. Other alterations were lower maximum angular velocity of the stifle and higher maximum angular velocity of the hip in G1 compared to G2, indicating respectively slower and faster flexion of these joints in G1. However, skin-marker movement may produce errors in the calculations of joint angles [1,2,8,39] representing a limitation. This should be considered especially in areas with more pronounced soft tissue coverage such as the stifle and hip joints [12,39].

## Conclusions

In conclusion, sheep of two different ages walking at a constant velocity present, within the same group, similar kinematic data between sides, and exhibit some differences in kinematic variables that may be age-related or body size. Further studies using sheep walking at similar Froude numbers are necessary to exclude the body size.
